# Total hip replacement: increasing femoral offset improves functional outcome

**DOI:** 10.1007/s00402-016-2527-4

**Published:** 2016-08-02

**Authors:** N. D. Clement, R. S. Patrick-Patel, D. MacDonald, S. J. Breusch

**Affiliations:** 1 Department of Orthopaedics and Trauma, Royal Infirmary of Edinburgh, Little France, Edinburgh, EH16 4SA UK; 2 University of Edinburgh, Little France, Edinburgh, EH16 4SB UK

**Keywords:** Hip, Arthroplasty, Offset, Outcome, Oxford hip score

## Abstract

**Introduction:**

The aim of this study was to assess the independent effect of radiographic measures of implant position, relative to pre-operative anatomical assessment, on the functional outcome of total hip arthroplasty according to change in the Oxford hip score (OHS) 1 year post surgery.

**Methods:**

A prospective cohort study was preformed to assess whether improvement in functional outcome (change in OHS at 1 year) and the relationship with femoral offset and length, and acetabular offset and height. After a power calculation 359 patients were recruited to the study and radiographic measures were performed by blinded observers. Regression analysis was used to assess the independent effect of the four radiographic measurements after adjusting for confounding variables.

**Results:**

There was a significant (*p* < 0.001) decrease in acetabular offset [5.3 mm, 95 % confidence interval (CI) 4.4–6.2] and increase in femoral offset (6.1 mm, 95 % CI 5.4–6.8). Hence there was no significant change in overall offset. Femoral offset was the only radiographic measure to be achieved statistical significance (*r* = 0.198, 95 % CI 0.063–0.333, *p* = 0.004) in relation to clinical outcome, with increasing offset being associated with a greater improvement in the OHS. On combining femoral and acetabular offset increasing offset was associated with a greater improvement in the OHS (*r* = 0.10, 95 % CI 0.01–0.19, *p* = 0.04).

**Conclusion:**

This study supports the long-held biomechanical theory of medialisation of the acetabular component with compensatory increased femoral offset results in improved functional outcome.

## Introduction

Total hip arthroplasty (THA) is one of the most successful surgical procedures performed, being named as the operation of the century [1], and is a cost effective procedure [2]. Despite the overall success of THA approximately 7–9 % of patients will be dissatisfied with their hip, 1 year after the surgery [3, 4]. The greatest predictor of patient satisfaction after surgery is improvement in their functional scores, both their hip specific Oxford Hip Score (OHS) and the Short Form (SF-) 12 physical component summary (PCS) score [3]. The main determinant of change in the OHS is the pre-operative score, with a worse score being associated with a greater improvement [5]. Other factors such as extremes of age, increasing body mass index (BMI), increasing comorbidity have also been associated with a diminished improvement in the OHS [5].

There is conflicting evidence as to whether leg length is a predictor of functional outcome and patient satisfaction after THA [6–8]. White and Dougall [6] conducted a prospective study to 200 patients, which concluded there was no correlation with the Harris hip score, SF-36 score or patient satisfaction with leg length post THA. More recently, Whitehouse et al. [8] affirmed these findings using the OHS, SF-12 score and satisfaction. In contrast to these studies other authors have reported that perceived leg length discrepancy significantly influences the OHS [9, 10] and patient satisfaction [7, 11] after THA.

There is, however, limited literature reporting the effect of implant position and reconstruction of centre of rotation and (femoral and acetabular) offset on the outcome of THA. Despite the theoretical biomechanical benefits of medializing the acetabular component and increasing femoral offset to compensate for this resulting in a more favourable moment arm [12, 13], there is limited literature to support any clinical effect. Studies by McGrory et al. [14] and Asayama et al. [15] demonstrated improved abductor muscle strength and a lower rate of Trendelenburg positive patients with increasing femoral offset, respectively. Judge et al. [5] demonstrated that female patients with an increased offset stem (exeter sizes 44 or more) had a significantly better outcome according to the OHS at 5 years when adjusting for confounding factors such as age, BMI, and pre-operative functional status, but whether this improved outcome was related to an absolute increase in the offset remains unknown.

The primary aim of this study was, therefore, to assess the independent effect of radiographic measures of implant position, relative to pre-operative anatomical assessment, on the functional outcome of THA according to change in the OHS 1 year post-surgery. The secondary aims were to assess the effect of radiographic measures of implant position on non-hip specific functional outcome (SF-12 and EuroQoL) scores and patient satisfaction 1 year post-surgery.

## Materials and methods

Ethical approval was obtained from the regional ethics committee (Research Ethics Committee, South East Scotland Research Ethics Service, Scotland, 11/AL/0079) for collection, analysis, and publication of the anonymised data.

During a 1 year period (2013) patients undergoing a THA at the study centre had functional outcome data recorded prospectively. Inclusion criteria for this study were: primary osteoarthritis, no deformity (precluding radiographic assessment), pre- and post-operative radiographs, and a cemented prosthesis. Patients undergoing revision during the first post-operative year were excluded. Patients undergoing consecutive bilateral THAs during the study period only had outcome and radiographic measures assessed for their first THA.

The patient demographics, comorbidities, BMI, and patient reported outcome measures were recorded at the pre-operative assessment clinic. Categories of comorbidity included were: heart disease, hypertension, lung disease, vascular disease, neurological problems, diabetes, stomach ulcer, kidney disease, liver disease, depression, back pain, and pain in other joints, which were all recorded as dichotomous variables.

The OHS [16], SF-12 score [17], and the EuroQoL were recorded pre-operatively and at 1 year post-operatively. The OHS consists of twelve questions assessed on a Likert scale with values from 0 to 4, a summative score is then calculated where 48 is the best possible score (least symptomatic) and 0 is the worst possible score (most symptomatic). The SF-12 is a generic assessment tool to measure a patient’s well-being, which is assessed using a PCS and a mental component summary (MCS) [17]. Both the SF-12 PCS and MCS range from 0 % (worst level of functioning) to 100 % (best level of functioning). EuroQoL (EQ) general health questionnaire evaluates five domains (-5D), which include: mobility, self-care, usual activities, pain/discomfort, and anxiety/depression [18]. In this study the EQ-5D-3L version of the EuroQoL questionnaire was used. This questionnaire assesses the five dimensions with the responses recorded at three levels of severity (no problems; some problems or extreme problems). An individual patient’s health state can be reported based on the five digit code for each domain, of which there are 243 possible health states. This index is on a scale of −1 to 1, where 1 represents perfect health and 0 represents death. Negative values represent a state perceived as worse than death.

Patient satisfaction was assessed by asking the question “How satisfied are you with your operated hip?” 1 year after surgery. The response was recorded using a five point Likert scale: very satisfied, satisfied, neutral, dissatisfied and very dissatisfied. Patients who recorded very satisfied or satisfied were classified as satisfied.

**Fig. 1:**
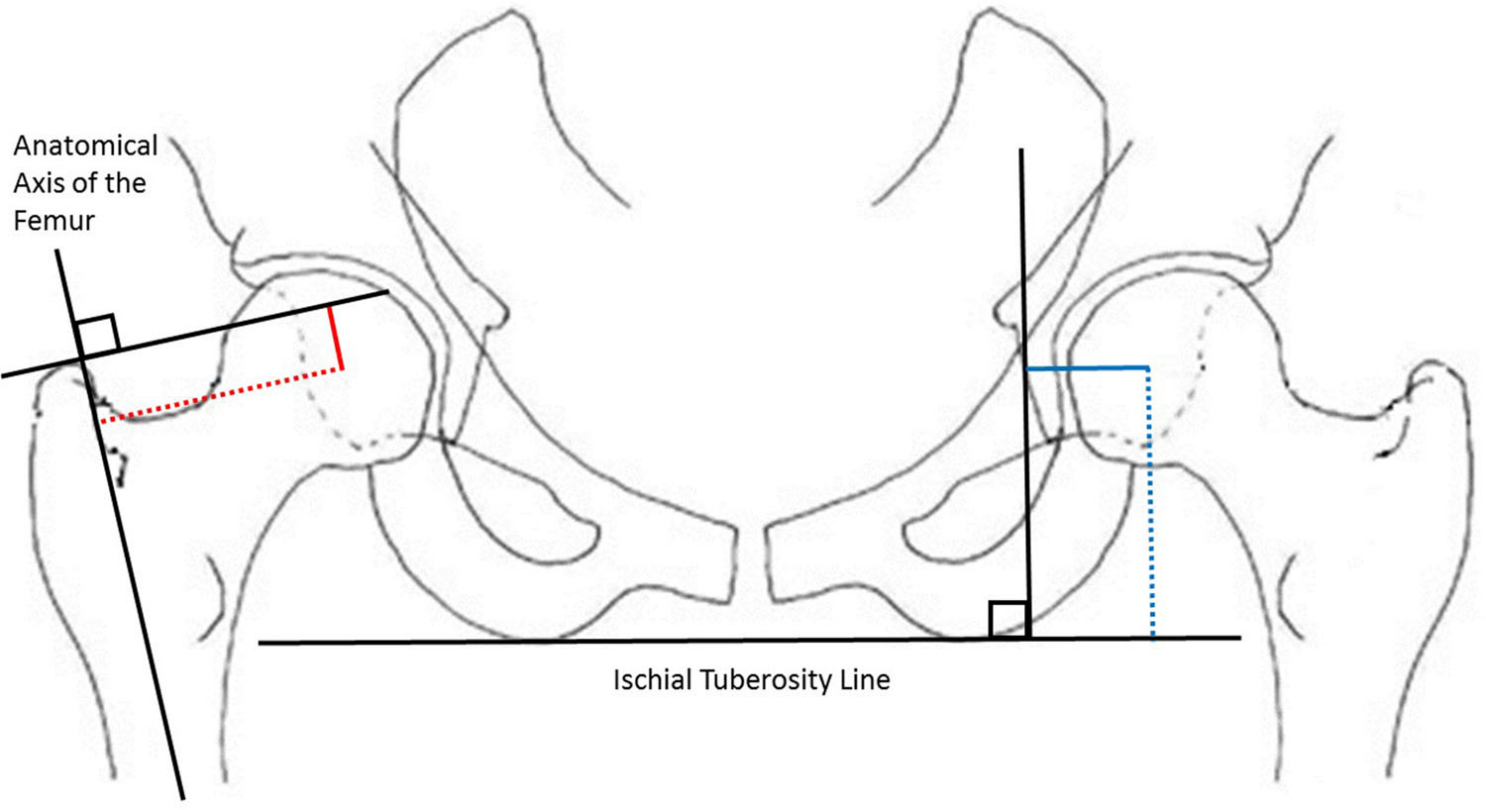
Diagrams defining the radiographic measurements-obtained [femoral offset (*red dashed line*) and length (*red line*), and acetabular offset (*blue line*) and height (*blue dashed line*)]

Radiographic assessment was performed by a blinded observer using a standardised protocol anterior–posterior radiograph of pelvis and hips pre-operatively and 1 year post-operatively. Radiographic measurement of offset and length for both the femoral and acetabular components were measured according to the methods described by Nunn et al. [19] and Jogger et al. [20] (Fig. 1). Loughead et al. [21] have demonstrated excellent inter- and intra-observer reliability/correlation of these radiographic measurements. Femoral offset was defined as the perpendicular distance from the anatomical axis of the femur to the centre of rotation of the femoral head. Femoral length was defined as the distance from the tip of the greater trochanter to a line perpendicular to the femoral head along the anatomical axis of the femur (Fig. 1). Acetabular offset was defined as the distance from the medial border of the teardrop to the centre of rotation of the acetabulum parallel to Hilgenreiner’s line. Cup height was defined as the distance from the centre of rotation of the acetabulum to a line drawn parallel with each ischial tuberosity, as this has been recently demonstrated to be more reliable than the inter-teardrop line [22]. All measurements were made using digital radiographs [Kodac© picture archiving and communication system (PACS) on a liquid crystal display] and the graphic measuring tools available. The measuring calibration tool was used to ensure equal measures were obtained. These measurements were repeated by a second observer for 30 cases to assess inter-observer variation and again for a further 30 patients to assess for intra-observer variation.

During the study period twelve consultant orthopaedic surgeons performed or supervised all included THAs. All patients underwent a THA using a cemented Exeter V40 (Stryker^®^) femoral stem (*n* = 327) or an Olympia (Biomet, Warsaw, Indiana) femoral stem (*n* = 32) using a Contemporary acetabular component (Stryker^®^). The surgical approach and technique were dependent upon surgeon preference with a higher prevalence of posterior approach (75.2 %, *n* = 270/359). All surgeons aimed to maintain offset and restore equal leg length, with a stable hip at the end of surgery. All patients received three peri-operative doses of prophylactic antibiotics (cefuroxime). A standardised rehabilitation protocol as per local clinical care pathway was used for all patients, with active mobilisation and full weight bearing on the first day post-operatively. Patients were then reviewed at 6 weeks, 6 months, and 12 months post-operatively as per local protocol.

Statistical analysis was performed using Statistical Package for Social Sciences version 17.0 (SPSS Inc., Chicago, IL, USA). Parametric and non-parametric tests were used as appropriate to assess continuous variables for significant differences between groups. A Student’s *t* test, unpaired and paired, was used to compare linear variables between groups. Pearson’s correlation was used to assess the relationship between linear variables. Multivariable linear regression analyses were used to identify independent predictors of outcome (change in the OHS). A single measure intraclass correlation coefficient (ICC) was used for the quantification of inter- and intra-observer reliability of the radiographic measurements. The values greater than 0.75 indicate satisfactory reliability [23]. A *p* value of <0.05 was defined as significant.

A power calculation was performed using data collected for the first 250 patients selected at random (pilot group). Using the OHS (primary outcome measure) and change in the radiographic measures, both of which demonstrated normal distribution, a significant (*p* = 0.001) correlation coefficient of 0.17 was demonstrated between the change in the femoral offset and the OHS. Using a bivariate normal model the required sample size using a two tailed analysis with an alpha of 0.05 and a power of 0.90 using the 0.17 correlation coefficient required 359 patients to be recruited.

## Results

During the study period 806 THA were performed at the study centre, of which 530 had pre- and post-operative outcome data recorded. For this study a cohort of 359 were randomly selected from the 530 patients with complete data met the inclusion criterion. There was no significant difference in gender (*p* = 0.87), age (*p* = 0.99), BMI (*p* = 0.92) or for the pre-operative functional measures (*p* > 0.80) between the study cohort and those patients not selected. The cohort demographics are presented in Table 1.

**Table 1:** Patient demographics for the study cohort (*n* = 359)

Demographic	Descriptive	*p* value
Gender (M/F) (*n*, % of cohort)	Male	139 (38.7)
	Female	220 (61.3)
Mean age (years: mean, SD)		67 (11.4)
Comorbidity (*n*, % of cohort)	Heart disease	36 (10.0)
	Hypertension	133 (37.0)
	Lung disease	20 (5.6)
	Diabetes mellitus	25 (7.0)
	Gastric ulceration	21 (5.8)
	Kidney disease	5 (1.4)
	Liver disease	1 (0.3)
	Anaemia	21 (5.8)
	Back pain	173 (48.2)
	Depression	48 (13.4)
BMI (kg/m^2^: mean, SD)		27.7 (5.7)

There was a significant improvement in the OHS, SF-12 PCS score and EQ-5D score at 1 year relative to pre-operative scores (Table 2). Six patients did not complete their satisfaction rating at 1 year. Three hundred and twenty-seven (92.6 %) patients declared their outcome as either very satisfied or satisfied, whereas 26 (7.4 %) thought their outcome was neutral, dissatisfied or very dissatisfied.

**Table 2:** Pre- and post-operative functional scores and the difference relative to pre-operative scores according to outcome measures assessed for the study cohort (*n* = 359)

Functional measure	Pre-operative (mean, SD)	Post-operative (mean, SD)	Difference	95 % CI	*p* value*
OHS	20.5 (8.3)	39.7 (8.8)	19.3	18.2 to 20.3	<0.0001
SF-12 PCS	31.8 (9.6)	45.0 (11.0)	13.2	11.8 to 14.6	<0.0001
SF-12 MCS	49.7 (12.3)	48.3 (8.8)	1.3	−0.03 to 2.70	0.06
EQ-5D	0.388 (0.313)	0.770 (0.259)	0.382	0.344 to 0.419	<0.0001

* Paired *t* test

**Fig. 2:**
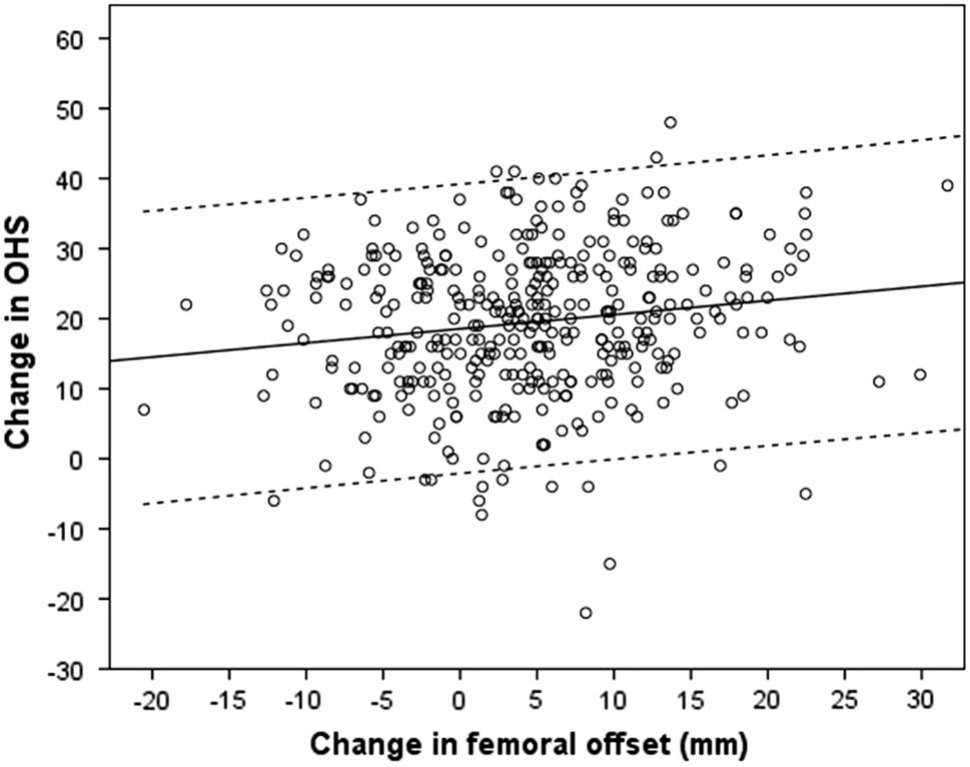
Correlation between changes in femoral offset and the OHS at 1 year post THA (*dashed line* represent 95 % confidence intervals)

The inter-observer variation for each of the radiographic measurements demonstrated satisfactory reliability (femoral offset ICC 0.91, femoral length ICC 0.86, acetabular offset ICC 0.93, and acetabular height ICC 0.87). The intra-observer variation demonstrated excellent reliability (femoral offset ICC 0.96, femoral length ICC 0.90, acetabular offset ICC 0.94, and acetabular height ICC 0.94).There were significant changes observed in femoral offset and length, and acetabular offset, but not for acetabular height (Table 3). There was a significant correlation demonstrated between change in femoral offset and change in the OHS at 1 year, with increasing femoral offset being associated with a greater improvement in the OHS (Fig. 2). A similar significant correlation with femoral offset was also observed with change in the SF-12 PCS, however no other radiographic measure achieved significant correlation with the other outcome measures assessed (Table 4). There was no significant difference between satisfied (*n* = 327) and dissatisfied (*n* = 26) patients for change in femoral offset (*p* = 0.81 *t* test) and length (*p* = 0.80 *t* test) or acetabular offset (*p* = 0.28 *t* test) and height (*p* = 0.47 *t* test).

**Table 3:** Pre- and post-operative radiographic measurements for the study cohort (*n* = 359)

Radiographic measures (mm)	Pre-operative (mean, SD)	Post-operative (mean, SD)	Difference	95 % CI	*p* value*
Femoral					
Offset	45.9 (9.3)	50.5 (7.7)	4.6	3.7 to 5.5	<0.001
Length	6.5 (7.9)	0.4 (6.5)	6.1	5.4 to 6.8	<0.001
Acetabular					
Offset	36.4 (8.8)	31.1 (5.7)	5.3	4.4 to 6.2	<0.001
Height	80.7 (9.2)	81.2 (9.9)	0.5	−0.14 to 1.1	0.13

* Paired *t* test

**Table 4:** Correlation of change of radiographic measurements with change in each of the outcome score at 1 year compared to pre-operative measures

Outcome measure	Radiographic measures			
	Femoral		Acetabular	
	Offset	Length	Offset	Height
OHS				
Correlation	0.160	0.052	0.049	0.007
*p* value*	**0.002**	0.33	0.36	0.90
SF-12 PCS				
Correlation	0.173	0.000	0.027	0.002
*p* value	**0.001**	0.99	0.61	0.97
SF-12 MCS				
Correlation	0.016	0.065	0.038	0.084
*p* value	0.78	0.22	0.48	0.11
EQ-5D				
Correlation	0.060	0.010	0.027	0.008
*p* value	0.26	0.86	0.61	0.88

* Pearson’s correlation

Bold *p* values represent significant correlations

Regression analysis was used to assess the independent effect of the four radiographic measures on change in the OHS when adjusting for confounding variables (Table 1). Femoral offset was the only radiographic measure to be achieved statistical significance (*r* = 0.198, 95 % CI 0.063–0.333, *p* = 0.004). Interestingly, on combing femoral and acetabular offset, increasing offset was associated with a greater improvement in the OHS (*r* = 0.10, 95 % CI 0.01–0.19, *p* = 0.04), but was not as significant as femoral offset independently. Hence, it would seem that overall offset is not as important as femoral offset.

## Discussion

A major limitation of this study was measuring offset using a digital radiograph of the pelvis. Measuring femoral offset using plane radiographic studies is limited by the precision of the technique and is dependent upon the patient position, magnification, and femoral rotation. It would have been of benefit to use Einbildröntgenanalyse (EBRA), which has been shown to have a measurement precision of around 0.8–1 mm [24, 25]. This methodology was not available due to local issues with compatibility with PACS. The most accurate method to measure offset is with a computer tomography (CT) scan [26]. To have obtained a CT for each patient pre- and post-operatively would not be clinically or ethically indicated [27]. However, results from three dimensional CT demonstrate similar average measures to plain radiographs [28, 29], and demonstrates good reliability [30]. Another limitation was the inclusion of several (12) different surgeons using differing surgical approaches (posterior or Hardinge) which may have influenced outcome and implant position. Furthermore, the study was underpowered (0.46) to demonstrate a significant difference in femoral offset between patients who were satisfied compared to those who were not (3.4 mm in this study), using the data from our cohort with a 12 to 1 (satisfied:dissatisfied) ratio, a cohort of 774 patients would have been required.

### Leg length

During THA it is estimated that on average the involved limb is lengthened by 2.5–6.2 mm [31], which is supported in the current study. Due to pre-operative shortening the affected limb is restored to within −1.0 to 3.5 mm of the contralateral side [31]. There is a large range of values for leg-length discrepancy post-THA and the threshold at which it becomes clinically important is controversial [32, 33]. Multiple authors have assessed numerous outcome measures such as energy consumption, change in gait mechanics, and joint pain. It is, however, considered that a discrepancy of less than 1 cm is acceptable and potentially as much as 2 cm of discrepancy is physiologically and subjectively tolerable by most adults but may be perceivable by the patient [31]. It is this perceived leg lengthening after THA that has been demonstrated to significantly influence the functional outcome according to the OHS [9, 10] and patient satisfaction [7, 11]. The current study demonstrated no correlation with limb length and outcome which is supported by the finding of Whitehouse et al. [8], who used the same outcome measures. Assessing outcome according to limb length after THA is difficult with potentially multiple factors affecting the outcome measure used, such as associated lower back pain [34], contralateral hip involvement, and stability of the hip. A stable THA is the ultimate goal and lengthening the limb to achieve this would seem to be tolerated up to 2 cm and should not influence the functional outcome or patient satisfaction.

### Offset

Charnley [12] and Muller [13] described the theoretical biomechanical benefits of medialising the acetabular component and increasing femoral offset to compensate some 45 years ago. McGrory et al. [14] demonstrated increased abductor power with medialisation of the acetabular component and increasing femoral offset, which was supported by Asayama et al. [15] with a lower rate of Trendelenburg positive patients with increasing femoral offset. The current study is novel, affirming the positive affect on functional outcome using the OHS with increasing femoral offset. This is supported by the study by Judge et al. [5], who found that patients with an increased offset stem (exeter 44 or more) had a significantly better outcome according to the OHS. However, increasing femoral offset has been associated with diminished pain relief after THA [28]. Lieds et al. [28] concluded, that those patients with the lowest offset had a significantly better outcome according to pain than those with increased offset (5 mm). However, they did not asses acetabular offset, which this study has done and demonstrated the overall offset (medial border of the teardrop to anatomical axis of the femur) did not significantly change. A biomechanical study assessing the effect of cup medialisation using finite element model according to CT analysis illustrated that the increase of the femoral offset may be effective in patients with less femoral anteversion, such that the patients gained more in terms of hip muscle moments [35]. However, medialisation of the acetabular component should balance against additional bone loss and potential proprioceptive implications of the non-anatomic centre of rotation. In addition, the joint reaction forces may increase and could influence the long-term survival of the THA [36], longer term studies would be needed to confirm or refute this. Over-increasing femoral and, hence, total offset may result in higher friction of the lateral trochanter and hence a higher rate of lateral hip pain, which would provide some plausible explanation for the findings by Lieds et al. [28]. Despite the positive results of the current study, further research is needed to determine the effect of changes of moment arms on function and joint reaction forces in the longer term.

Computer navigation has been shown to enable quantitative control of offset, both femoral and acetabulum during THA, with the centre of hip rotation being maintained within 5 mm of the contralateral normal side [37]. This technology could be used to assess the amount of medial displacement of the acetabular component and increased femoral offset intra-operatively. This could, then, be used to assess and quantify the optimal offset of both components that would facilitate functional outcome and longevity with minimisation of wear. Our study has failed to demonstrate that limb length, measured by femoral length and acetabular height are related to functional outcome. In contrast, we demonstrated that decreasing femoral (and hence overall) offset, measured radiographically, is independently associated with poorer hip specific function according to the OHS. Interestingly, despite the improved OHS with increased femoral offset there was no significant improvement in patient satisfaction at 1 year with the THA. Although there was a significant increase in the femoral offset (5 mm), this was associated with a significant decrease in acetabular offset (5 mm) due to medialisation with no change in the overall offset. Hence one could argue that the desired cup medialisation must be compensated for by increasing femoral offset, with the ultimate goal of not ending up with a reduced overall/combined offset. This is the most important finding and conclusion from our study and, therefore, has implications on pre-operative planning. Our data suggest that a number of commercially available femoral stem designs may not allow for this unless lateralised stem designs are available.

This study supports the long-held biomechanical theory of medialisation of the acetabular component with compensatory increased femoral offset results in improved functional outcome, which has been demonstrated using a hip-specific validated outcome measure. The exact anatomic parameters of the femoral and acetabular components that relate to the optimal outcome of patients undergoing a THA remain to be identified. Potentially computer navigation may help improve implant positing and attain an optimal component orientation that achieves a stable THA with maximal functional outcome and longevity for each patient.
